# Secondary Shiga Toxin–Producing *Escherichia coli* Infection, Japan, 2010–2012

**DOI:** 10.3201/eid2212.160783

**Published:** 2016-12

**Authors:** Tomoko Morita-Ishihara, Sunao Iyoda, Atsushi Iguchi, Makoto Ohnishi

**Affiliations:** National Institute of Infectious Diseases, Tokyo, Japan (T. Morita-Ishihara, S. Iyoda, M. Ohnishi);; University of Miyazaki, Miyazaki, Japan (A. Iguchi)

**Keywords:** Shiga toxin–producing Escherichia coli, STEC, healthy adults, asymptomatic carriers, incidence rate, secondary transmission, bacteria

## Abstract

To evaluate the potential public health risk caused by secondary Shiga toxin–producing *Escherichia coli* (STEC) infections in Japan, we investigated the prevalence and characteristics of STEC isolated from healthy adults during 2010–2012. Although prevalence among healthy adults was high, most STEC organisms displayed characteristics rarely found in isolates from symptomatic patients.

Shiga toxin–producing *Escherichia coli* (STEC), which is characterized by production of the Shiga toxin (Stx) or possession of the Stx-encoding genes, is a notable human pathogen that causes diarrhea, hemorrhagic colitis, and hemolytic uremic syndrome (*1*,*2*). In Japan, STEC infection is a notifiable disease, and ≈3,500–4,500 (2.7–3.5/100,000 population) annual cases of STEC infection (including asymptomatic carriers) have been reported since 2006 (*3**–**5*). For ≈50%–60% of these notifiable cases, serotype and Stx type of laboratory-confirmed STEC isolate and clinical manifestations associated with STEC infection are reported (*3*). To date, most STEC isolates from asymptomatic carriers collected in Japan were isolated during laboratory-based investigation of outbreaks or intrafamilial infections, together with those from sporadic patients. Therefore, we believe that the characteristics of such STEC isolates could be similar to those from ill patients. We conducted a large-scale study to investigate the prevalence and characteristics of STEC isolated from healthy adults in Japan to evaluate the potential public health risk of infection due to secondary STEC infection. 

## The Study

A total of 2,774,824 fecal samples were collected from 472,734 healthy adults throughout Japan during April 2010–March 2012; these samples were examined at Japan Microbiological Laboratory Co., Ltd. (Sendai, Japan) (Technical Appendix). These healthy persons included food handlers and those who worked in childcare and eldercare facilities; each person was tested >1 times over 2 years. When >1 STEC isolate from the same patient had the same serotype and virulence factor type, we used the first detected isolate in this study. Of 472,734 healthy adults examined, 398 (0.08%) were positive for STEC. Therefore, the estimated incidence rate of asymptomatic carriers among healthy adults was 84.2/100,000 population, indicating that asymptomatic STEC infections are highly prevalent among healthy adults.

A total of 399 STEC organisms were isolated from 398 healthy adults. O-serogrouping showed that 339 isolates comprised 61 different O serogroups; 60 isolates were O serogroup untypeable (Technical Appendix Table). Two isolates obtained from the same person >12 months apart belonged to serogroups O103 (first isolate) and O91 (second isolate). The dominant O serogroup of isolates from healthy adult carriers was O91 (n = 89, 22.3%), followed by O103 (n = 23, 5.8%). Of STEC infection cases associated with patients reported during 2010–2011 in Japan, 97.4% were caused by either STEC O157 (68.8%), O26 (16.9%), O111 (3.9%), O145 (3.1%), O103 (2.8%), or O121 (1.9%) isolates (*4*,*5*). In this study, STEC O157 (n = 13), O26 (n = 6), O145 (n = 2), O103 (n = 23), and O121 (n = 1) isolates were detected, but STEC O111 isolates were not (Technical Appendix Table). Therefore, the STEC isolates belonging to these 6 O serogroups represented only 11.3% of all isolates from healthy adults. These results show that the prevalence of O serogroups among STEC from healthy adults was clearly different from that among symptomatic patients.

We also determined the *stx* type of STEC isolates and investigated the presence of virulence factors (Technical Appendix). Of 399 STEC isolates, 201 (50.4%) harbored the *stx1* gene only (*stx1* type), 160 (40.1%) harbored the *stx2* gene only (*stx2* type), and 38 (9.5%) harbored both *stx1* and *stx2* genes (*stx1/stx2* type) (Table 1). Adherence factors that contribute to virulence of STEC, *eae*, *saa*, and Eib, were detected in 55 (13.8%), 125 (31.3%), and 102 (25.6%) isolates, respectively (Table 1) (*6*–*8*). No STEC isolate harbored >2 of these adherence genes/factors; *aggR* was not detected in any isolates; and 117 (29.3%) isolates did not contain any adherence genes/factors.

**Table 1:** Serogroups, *stx* genes, and virulence genes of Shiga toxin–producing *Escherichia coli* isolates from healthy adults, Japan, 2010-–2012

Serogroup	No. isolates	No. isolates that possessed each gene type					
		*stx1*	*stx1/stx2*	*stx2*	*eae*	*saa*	Eib
O91	89	84	5			11	77
O103	23	23			22		
O8	15	1		14		2	
O113	15		2	13		10	
O128	15	4	8	3		8	5
O110	13			13			
O157	13	1	4	8	13		
O146	10	2	4	4		8	1
O174	9		1	8		1	
O100	8			8		1	
O181	8	2		6		7	
O55	7	7				1	
O76	7	7			1	5	
O26	6	6			6		
O74	6			6		6	
O112	5			5		1	2
O115	5	5					
O183	5	4	1			3	
O93	4	3		1		2	
O156	4	4			4		
O163	4	2		2		1	
O186	4	2	2			4	
O38	3		1	2		3	
O78	3	2	1			3	
O159	3			3			
O166	3			3		2	
O168	3			3			
O171	3	1		2		1	
O6	2	1		1		1	
O18	2	1		1			1
O22	2	1		1		1	
O25	2			2			
O28	2			2		2	
O87	2	1		1			
O101	2			2			
O109	2	2				1	
O119	2	2				2	
O130	2			2		2	
O145	2	1	1		2		
O150	2	2				2	
O178	2	1		1		2	
O2	1			1			
O5	1		1				1
O15	1			1			
O53	1			1			
O65	1	1				1	
O75	1		1			1	
O77	1	1					
O82	1	1				1	
O96	1		1			1	
O105	1			1		1	
O108	1	1				1	
O117	1	1					
O121	1			1	1		
O136	1	1					
O137	1	1				1	
O149	1		1			1	
O175	1			1			
O177	1	1			1		
O179	1			1		1	
O185	1			1			
O untypeable	60	21	4	35	5	23	15
Total	399	201	38	160	55	125	102

The combination of *stx2* and *eae* genes in STEC is considered a risk factor for high virulence of STEC because these 2 genes are often found together in STEC isolates associated with severe disease such as hemolytic uremic syndrome (*9*). To investigate the prevalence of such risk factors among STEC isolates from healthy adults, we analyzed *eae*-positive isolates (n = 55). In this study, all STEC isolates belonging to 1 of 6 major O serogroups (O157:H7/H-, O26:H11, O111:H-, O103:H2/H11, O145:H-, O121:H19) frequently found in patient-derived isolates were positive for the *eae* gene, except for an O103:H2 isolate (Table 2; Technical Appendix). O156:H-/HUT (n = 4), O76:H7 (n = 1), O177:H- (n = 1), and O-untypeable (n = 5) isolates were also positive for the *eae* gene. From analysis of *stx1*, *stx2*, and their subtypes, 16 of 55 *eae*-positive isolates harbored the *stx2* gene; their *stx2* subtypes were *stx2a* (n = 5), *stx2c* (n = 10), and *stx2e* (n = 1) (Table 2; Technical Appendix Table). *stx2a* and *stx2c* were the *stx2* subtypes most commonly found in patient-derived isolates (*10*,*11*). The STEC isolates harboring both *eae* and *stx2* occupied 4% of all STEC isolates from healthy adults. Therefore, we estimated that the incidence rate of healthy asymptomatic carriers infected with STEC isolates harboring both *eae* and *stx2* was 3.4/100,000 population (16/472,734 isolates). These results highlight the potential risk of serious STEC infection, which may be transmitted by secondary transmission from asymptomatic carriers.

**Table 2:** Serotypes and *stx* types of *eae*-positive Shiga toxin–producing *Escherichia coli* isolates from healthy adults, Japan, 2010–2012

Serotype		No. *eae*-positive isolates*	No. *stx*-positive isolates†		
			*stx1*	*stx1/stx2*	*stx2*
O103:	H2	21	21		
	H11	1	1		
O157:	H7	10		2 (*stx*2a)	8 (*stx*2a [1], *stx*2c [7])
	H-	3	1	2 (*stx*2c)	
O26:	H11	6	6		
O156:	H-	2	2		
	HUT	2	2		
O145:	H-	2	1	1 (*stx*2a)	
O76:	H7	1	1		
O121:	H19	1			1 (*stx*2a)
O177:	H-	1	1		
O	Yntypeable	5	3		2 (*stx*2c [1], *stx*2e [1])
Total		55	39	5	11

*The number of *eae*-negative isolates among the same serotype: O103:H2 (1), O76:H19 (5), O76:H- (1). †The name of the positive subtype is in parentheses; the number subtype-positive isolates are in brackets.

## Conclusions

We found that the incidence rate of STEC infection in healthy asymptomatic carriers was 84.2/100,000 population. This finding suggests that the risk of secondary transmission to susceptible persons may be higher than originally thought. However, many STEC isolates from healthy adults belong to O serogroups that are rarely found in STEC isolates from symptomatic patients, and >80% of those isolates did not have the *eae* gene that is frequently detected in STEC isolates from symptomatic patients.

Recently, STEC outbreaks caused by isolates from 6 major O serogroups (O157:H7/H-, O26:H11, O111:H-, O103:H2/H11, O145:H-, O121:H19) have been frequently reported in childcare facilities in Japan (*12*). Person-to-person transmission is considered a major route of infection in such outbreaks, and many adult family members identified in such outbreaks had asymptomatic cases (*12*). In addition, those who work within child- and elder-care facilities are possibly more likely to be exposed to STEC organisms than are the general population because STEC organisms are more likely to be shed in higher numbers in children and elders from such facilities (*13*). We found that STEC O157:H7/H-, O26:H11, O103:H2, O121:H19, and O145:H- were isolated from asymptomatic carriers. These findings suggest that a portion of STEC infection due to these serotypes may be caused by secondary transmission through asymptomatic carriers.

Although the prevalence of STEC O157, O26, and O111 in retail raw foods is monitored by the National Food Surveillance System in Japan, prevalence of STEC belonging to other O serogroups in foods is unknown. In 1 study regarding STEC strains from food-producing animals in Japan during 1999–2001, of the bovine isolates, 30.6% belonged to serotypes frequently implicated in human disease, and 37% harbored the *eae* gene (*14*). Isolates with such serotypes and *eae* were not found among the isolates from swine (*14*). Many STEC isolates from food-producing animals, as with those from healthy adults, displayed characteristics rarely found in patient-derived isolates (*14*). Food handlers may be at a greater risk of STEC transmission through food preparation, because they are more likely to be exposed to organisms from food-producing animals than the general population (*15*).

Our findings provide scientific evidence that can be useful in the management of STEC infection, in particular, in detecting asymptomatic carriers in Japan. Such identification could result in a decrease in asymptomatic carriers and in secondary transmission of STEC organisms.

Technical Appendix Material and methods of study of secondary Shiga toxin–producing *Escherichia coli* infection (STEC), Japan, 2010–2012, including a table showing *stx*2 subtypes of STEC isolates.

## References

[1] Paton JC, Paton AW. Pathogenesis and diagnosis of Shiga toxin-producing *Escherichia coli* infections. Clin Microbiol Rev. 1998;11:450–79. 9665978 10.1128/cmr.11.3.450 PMC88891

[2] Nataro JP, Kaper JB. Diarrheagenic *Escherichia coli.* Clin Microbiol Rev. 1998;11:142–201. 9457432 10.1128/cmr.11.1.142 PMC121379

[3] National Institute of Infectious Diseases, Ministry of Health, Labour and Welfare, Japan. Pathogen surveillance system in Japan and Infectious Agents Surveillance Report (IASR). Infect Agents Surveill Rep. 2010;31:69–70. http://idsc.nih.go.jp/iasr/31/361/tpc361.html

[4] National Institute of Infectious Diseases, Ministry of Health, Labour and Welfare, Japan. Enterohemorrhagic *Escherichia coli* infection in Japan as of April 2011. Infect Agents Surveill Rep. 2011;32:125–7.

[5] National Institute of Infectious Diseases, Ministry of Health, Labour and Welfare, Japan. Enterohemorrhagic *Escherichia coli* infection in Japan as of April 2012. Infect Agents Surveill Rep. 2012;33:115–7. http://www.niid.go.jp/niid/en/iasr-vol33-e/865-iasr/2134-tpc387.html

[6] Jerse AE, Yu J, Tall BD, Kaper JB. A genetic locus of enteropathogenic *Escherichia coli* necessary for the production of attaching and effacing lesions on tissue culture cells. Proc Natl Acad Sci U S A. 1990;87:7839–43. 10.1073/pnas.87.20.7839 2172966 PMC54845

[7] Lu Y, Iyoda S, Satou H, Satou H, Itoh K, Saitoh T, et al. A new immunoglobulin-binding protein, EibG, is responsible for the chain-like adhesion phenotype of locus of enterocyte effacement-negative, shiga toxin-producing *Escherichia coli.* Infect Immun. 2006;74:5747–55. 10.1128/IAI.00724-06 16988252 PMC1594913

[8] Paton AW, Srimanote P, Woodrow MC, Paton JC. Characterization of Saa, a novel autoagglutinating adhesin produced by locus of enterocyte effacement-negative Shiga-toxigenic *Escherichia coli* strains that are virulent for humans. Infect Immun. 2001;69:6999–7009. 10.1128/IAI.69.11.6999-7009.2001 11598075 PMC100080

[9] Ethelberg S, Olsen KE, Scheutz F, Jensen C, Schiellerup P, Enberg J, et al. Virulence factors for hemolytic uremic syndrome, Denmark. Emerg Infect Dis. 2004;10:842–7. 10.3201/eid1005.030576 15200817 PMC3323205

[10] Orth D, Grif K, Khan AB, Naim A, Dierich MP, Würzner R. The Shiga toxin genotype rather than the amount of Shiga toxin or the cytotoxicity of Shiga toxin in vitro correlates with the appearance of the hemolytic uremic syndrome. Diagn Microbiol Infect Dis. 2007;59:235–42. 10.1016/j.diagmicrobio.2007.04.013 17931818

[11] Persson S, Olsen KE, Ethelberg S, Scheutz F. Subtyping method for *Escherichia coli* shiga toxin (verocytotoxin) 2 variants and correlations to clinical manifestations. J Clin Microbiol. 2007;45:2020–4. 10.1128/JCM.02591-06 17446326 PMC1933035

[12] Kanayama A, Yahata Y, Arima Y, Takahashi T, Saitoh T, Kanou K, et al. Enterohemorrhagic *Escherichia coli* outbreaks related to childcare facilities in Japan, 2010-2013. BMC Infect Dis. 2015;15:539–46. 10.1186/s12879-015-1259-3 26589805 PMC4654900

[13] Cornick NA, Jelacic S, Ciol MA, Tarr PI. *Escherichia coli* O157:H7 infections: discordance between filterable fecal shiga toxin and disease outcome. J Infect Dis. 2002;186:57–63. 10.1086/341295 12089662

[14] Kijima-Tanaka M, Ishihara K, Kojima A, Morioka A, Nagata R, Kawanishi M, et al. A national surveillance of Shiga toxin-producing *Escherichia coli* in food-producing animals in Japan. J Vet Med B Infect Dis Vet Public Health. 2005;52:230–7. 10.1111/j.1439-0450.2005.00852.x 16115097

[15] Doyle MP. *Escherichia coli* O157:H7 and its significance in foods. Int J Food Microbiol. 1991;12:289–301. 10.1016/0168-1605(91)90143-D 1854598

