# Supplementary material for: Secondary Shiga Toxin–Producing Escherichia coli Infection, Japan, 2010–2012
Source: Emerg Infect Dis. 2016 Dec;22(12):2181–4. doi: 10.3201/eid2212.160783 (PMC5189154; doi:10.3201/eid2212.160783)
Supplement: Technical Appendix — Material and methods of study of secondary Shiga toxin–producing Escherichia coli infection (STEC), Japan, 2010–2012, including a table showing stx2 subtypes of STEC isolates. [file 16-0783-Techapp-s1.pdf]

Article DOI: http://dx.doi.org/10.3201/eid2212.160783

Secondary Shiga Toxin–Producing *Escherichia coli* Infection, Japan, 2010–2012

Technical Appendix

Materials and Methods

Clinical Samples

Fecal samples were collected from healthy adults throughout Japan from April 2010 to March 2012. A total of 2,774,824 stool samples from 472,734 healthy adults including food handlers and workers in daycare centers for children and elders were examined at Japan Microbiological Laboratory Co., Ltd (Sendai, Japan). These persons are required by law to undergo periodic fecal examination. Stools were streaked on to Drigalski agar plates (Kyokuto Seiyaku, Tokyo, Japan) and enterohemolysin blood agar plates (Kanto Chemical, Tokyo, Japan), before overnight incubation at 37°C. From grown colonies, 5 lactose-fermenting colonies or hemolytic colonies were tested by PCR for *stx1* and *stx2*. *stx*-positive colonies were confirmed biochemically as *Escherichia coli* by using appropriate sugars and other metabolites and API 20E (bioMérieux, Durham, NC, USA).

Serotyping of *E. coli*

Typing of O and H antigens was performed by the standard agglutination test by using commercially available *E. coli* antisera (Denka Seiken, Tokyo, Japan; Statens Serum Institute,

Copenhagen, Denmark). *E. coli* O-genotyping PCR was used as a supplementary tool for O serogroup identification (*1*).

Detection of *stx1*, *stx2*, and Their Subtypes

Detection of *stx1* and *stx2* was performed by PCR with primers LP30/LP31 and LP43/LP44 (*2*) and primers vtx1-det-F1/vtx1-det-R1, F4/R1 and F4-f/R1-e/f (*3*). Subtyping of *stx2* was performed as described (*3*). *E. coli* O157:H7 strain Sakai was used as a positive control strain for *stx1*, *stx2* and *eae*. Seven *E. coli* strains were used as positive control strains for the 7 subtypes of *stx2* as described (*3*).

Detection of Virulence Genes/Factors

We used PCR to detect *eae*, *saa*, and *aggR* as described in the following articles: *saa* (*4*); *eae* (*5*); and *aggR* (*6*). Eib was detected by using an immunodetection assay with human-derived IgG Fc- and IgA-conjugated with horseradish peroxidase (HRP; Jackson Immunoresearch Laboratories) on centrifuged bacterial cell pellets from overnight cultures, as described previously (*7*). Three *E. coli* strains were used as positive control strains to detect *saa* (*4*), *aggR* (*6*), and Eib (*7*).

References

1. Iguchi A, Iyoda S, Seto K, Morita-Ishihara T, Scheutz F, Ohnishi M; Pathogenic *E. coli* Working Group in Japan. *Escherichia coli* O-genotyping PCR: a comprehensive and practical platform for molecular O serogrouping. J Clin Microbiol. 2015;53:2427–32. PubMed http://dx.doi.org/10.1128/JCM.00321-15

2. Cebula TA, Payne WL, Feng P. Simultaneous identification of strains of *Escherichia coli* serotype O157:H7 and their Shiga-like toxin type by mismatch amplification mutation assay-multiplex PCR. J Clin Microbiol. 1995;33:248–50. PubMed

3. Scheutz F, Teel LD, Beutin L, Piérard D, Buvens G, Karch H, et al. Multicenter evaluation of a sequence-based protocol for subtyping Shiga toxins and standardizing Stx nomenclature. J Clin Microbiol. 2012;50:2951–63. PubMed http://dx.doi.org/10.1128/JCM.00860-12

4. Paton AW, Paton JC. Direct detection and characterization of Shiga toxigenic *Escherichia coli* by multiplex PCR for *stx1*, *stx2*, *eae*, *ehxA*, and *saa*. J Clin Microbiol. 2002;40:271–4. PubMed http://dx.doi.org/10.1128/JCM.40.1.271-274.2002

5. Narimatsu H, Ogata K, Makino Y, Ito K. Distribution of non-locus of enterocyte effacement pathogenic island-related genes in *Escherichia coli* carrying *eae* from patients with diarrhea and healthy individuals in Japan. J Clin Microbiol. 2010;48:4107–14. PubMed http://dx.doi.org/10.1128/JCM.00677-10

6. Ito K, Matsushita S, Yamazaki M, Moriya K, Kurazono T, Hiruta N, et al. Association between aggregative adherence fimbriae types including putative new variants and virulence-related genes and clump formation among aggR-positive *Escherichia coli* strains isolated in Thailand and Japan. Microbiol Immunol. 2014;58:467–73. PubMed http://dx.doi.org/10.1111/1348-0421.12173

7. Lu Y, Iyoda S, Satou H, Satou H, Itoh K, Saitoh T, et al. A new immunoglobulin-binding protein, EibG, is responsible for the chain-like adhesion phenotype of locus of enterocyte effacement-negative, shiga toxin-producing *Escherichia coli*. Infect Immun. 2006;74:5747–55. PubMed http://dx.doi.org/10.1128/IAI.00724-06

Technical Appendix Table. stx2 subtypes of STEC isolates from healthy adults

Serogroup	No. *stx2*-positive isolates	No. isolates with *stx2* subtype: a	b	c	d	e	g	combination of *stx2* subtypes (no. of isolates)
O113	15	8	2	7	12			a+c+d (5), a+d (2), c+d (2)
O8	14	3		2	3	9		a+d (2), c+d (1)
O110	13	13	1		1			a+b+d (1)
O157	12	3		9				
O128	11		10		1			
O174	9	5	2	3	2	1		a+c+d (2)
O100	8					8		
O146	8		8					
O74	6	5			1			
O181	6	6	1		1			a+b (1), a+d (1)
O91	5	2	5					a+b (2)
O112	5		2	3	4			b+d (2), c+d (2)
O38	3	2	1					
O159	3	3			3			a+d (3)
O166	3	2		1	1			c+d (1)
O168	3				2		1	
O25	2	2			2			a+d (2)
O28	2	2		1				a+c (1)
O101	2					2		
O130	2	2						
O163	2				2			
O171	2	1		1				
O186	2		2					
O2	1				1			
O5	1		1					
O6	1	1		1	1			a+c+d (1)
O15	1						1	
O18	1						1	
O22	1			1				
O53	1	1			1			a+d (1)
O75	1		1					
O78	1		1					

Serogroup	No. *stx2*-positive isolates	No. isolates with *stx2* subtype: a	b	c	d	e	g	combination of *stx2* subtypes (no. of isolates)
O87	1		1					
O93	1	1						
O96	1	1						
O105	1				1			
O121	1	1						
O145	1	1						
O149	1		1					
O175	1				1			
O178	1	1						
O179	1	1						
O183	1				1			
O185	1			1				
OUT	39	15	12	3	5	9	1	a+b+c+d (1), a+c+d (1), b+e (1)
Total	198	82	51	33	46	29	4	35
